# Hantaviruses in São Paulo State, Brazil

**DOI:** 10.3201/eid0907.030087

**Published:** 2003-07

**Authors:** Luiz T.M. Figueiredo, Marcos L. Moreli, Gelse M. Campos, Ricardo L.M. Sousa

**Affiliations:** * University of São Paulo, São Paulo, Brazil

**To the Editor**: Hantavirus pulmonary syndrome (HPS) is an emerging health problem in Brazil. This syndrome was first reported in 1993 in three persons living in a rural area of Juquitiba County; two of them died of acute respiratory failure (*1*). Although Juquitiba County is part of the metropolitan area of greater São Paulo City, patients lived in a recently deforested region. From 1993 through 2002, approximately 200 HPS cases were reported in Brazil, with a 40% case-fatality ratio (Ministry of Health of Brazil, Report on Hantavirus cases 1993–2002, unpub. data).

The wild rodent *Bolomys laziurus* is believed to be the most important hantavirus reservoir in the State of São Paulo, based on high levels of specific antibodies observed in serum from captured specimens (L.E. Pereira, Adolpho Lutz Institute, pers. comm., 2001). The economy of the inland region of Ribeirão Preto in the State of São Paulo, with its 3.5 million inhabitants, is based on the sugar cane agroindustry. The region has been almost completely deforested, with important consequences to the environment and wild rodent ecology. Twenty HPS cases were reported in Ribeirão Preto in the last 5 years, with a 60% case-fatality ratio. Review of medical records showed that a prodromic fever occurred in all 14 case-patients studied; dyspnea, cough, hypotension, and tachycardia occurred in about two thirds of patients; and hemorrhagic phenomena (hematuria, melena, and hypermenorrhea) in about one third. Thrombocytopenia was observed in all the patients, elevated hematocrit in about three fourths, and leukocytosis with neutrophilia and a left shift in the differential count in about two thirds. Serum creatinine levels were also increased (average level 2 mg/dL). Chest radiographs showed diffuse alveolar flocculant infiltrates in most cases (*2*,*3*). Laboratory diagnosis of HPS was made by serologic testing (enzyme-linked immunosorbent assay [ELISA]) in 18 cases and by reverse transcription–polymerase chain reaction (RT-PCR) in 11 cases; for 7 cases, both techniques were used. We performed a nucleotide sequence analysis of the N gene of hantavirus (residues 236–477) obtained from the blood of 11 of the 20 patients. This analysis showed that the infections were caused by Araraquara virus, a previously known hantavirus that had been detected by RT-PCR in the serum of an HPS patient living in a nearby county (*4*). Thus, Araraquara virus is the causative agent of a severe form of HPS, with a high death rate. This high death rate could also be related to the lack of adequate initial therapy provided by clinicians who probably did not immediately suspect HPS and may have not recommended hospitalization in intensive-care units. In addition, some hospitalized patients were in shock when first seen and were rehydrated with massive quantities of fluids, which may have aggravated pulmonary edema and contributed to death.

The occurrence of 10% of the Brazilian HPS reported cases in Ribeirão Preto indicates that this region is suitable for studying the epidemiology of hantavirus infections. A serologic survey conducted in the region in 1999, which included 567 primary-care patients from Ribeirão Preto, Guariba, and Jardinópolis Counties, found that 7 (1.23%) of them had immunoglobulin (Ig) G antibodies to Sin Nombre virus by ELISA and that 5 of those lived in Jardinópolis (population 30,000), a county where a fatal case of HPS occurred in 1999 (*5*). Thus, Jardinópolis County was chosen for a population-based survey. In May 2001, we obtained personal information and collected fingerprick blood samples from 818 participants, 15–70 years of age, living in urban and rural areas of the county. IgG antibodies to the N recombinant protein of Andes virus were detected by ELISA in the blood samples of 14.3% of the participants (*5*). Even though all HPS cases in Ribeirão Preto were associated with rural activity and rodent exposure, these serologic data suggest that hantavirus infections are common in Jardinópolis County, independent of sex, profession, or history of contact with rodents. None of the 14.3% participants with IgG antibodies to hantavirus had a history of HPS-like disease, and the ELISA test showed cross-reactions with most of the South American hantaviruses, including Araraquara. Persons living in the urban area had higher levels of antibodies to hantavirus than those from rural areas. In Ribeirão Preto, the physical boundaries of cities have expanded to incorporate other areas, encroaching upon rural areas with many popular subsidized housing complexes. Work-related and recreational rural activities in that region are also frequent, which makes it difficult to interpret these data. These results suggest that in this region of southeast Brazil, hantaviruses may be causing undiagnosed asymptomatic or clinically minor infections in addition to typical HPS. This finding envokes important questions. Is more than one hantavirus circulating in this region, causing mostly benign infections? Is Araraquara virus widespread, causing mostly inapparent infections and only rarely causing HPS? Would HPS be associated with some predisposing condition in the infected person? If more than one hantavirus is circulating in the region, could urban rodents be reservoirs?

Further studies are necessary to better understand the epidemiology and clinical signs and symptoms of hantavirus infection in the region of Ribeirão Preto. Such studies should emphasize determining the reservoirs, the modes of virus transmission to people, and the possible distinct clinical forms of hantavirus infections.
